# PrEP uptake and early persistence among adolescent girls and young women receiving services via community and hybrid community-clinic models in Namibia

**DOI:** 10.1371/journal.pone.0289353

**Published:** 2023-08-30

**Authors:** Gena Barnabee, Idel Billah, Lylie Ndeikemona, Lukas Silas, Alison Ensminger, Ellen MacLachlan, Abigail K. Korn, Susan Mawire, Christa Fischer-Walker, Laimi Ashipala, Norbert Forster, Gabrielle O’Malley, Jennifer Velloza

**Affiliations:** 1 International Training and Education Center for Health, Department of Global Health, University of Washington, Seattle, WA, United States of America; 2 International Training and Education Center for Health, Department of Global Health, University of Washington, Windhoek, Namibia; 3 Ministry of Health and Social Services, Windhoek, Namibia; 4 Star for Life, Windhoek, Namibia; 5 FHI360, Durham, NC, United States of America; 6 Department of Epidemiology & Biostatistics, University of California San Francisco, San Francisco, CA, United States of America; Pan American Health Organization, UNITED STATES

## Abstract

**Introduction:**

Adolescent girls and young women (AGYW) face barriers in accessing clinic-based HIV pre-exposure prophylaxis (PrEP) services and community-based models are a proposed alternative. Evidence from such models, however, is limited. We evaluated PrEP service coverage, uptake, and early persistence among AGYW receiving services through community and hybrid models in Namibia.

**Methods:**

We analyzed routine data for AGYW aged 15–24 who initiated PrEP within HIV prevention programming. PrEP was delivered via three models: community-concierge (fully community-based services with individually-tailored refill locations), community-fixed (community-based initiation and refills delivered by community providers on a set schedule at fixed sites), and hybrid community-clinic (community-based initiation and referral to clinics for refills delivered by clinic providers). We examined proportions of AGYW engaged in services along a programmatic PrEP cascade, overall and by model, and assessed factors associated with PrEP uptake and early persistence (refill within 15–44 days after initiation) using multivariable generalized estimating equations.

**Results:**

Over 10-months, 7593 AGYW participated in HIV prevention programming. Of these, 7516 (99.0%) received PrEP education, 6105 (81.2%) received HIV testing services, 6035 (98.9%) tested HIV-negative, and 2225 (36.9%) initiated PrEP. Of the 2047 AGYW expected for PrEP refill during the study period, 254 (12.4%) persisted with PrEP one-month after initiation. Structural and behavioral HIV risk factors including early school dropout, food insecurity, inconsistent condom use, and transactional sex were associated with PrEP uptake. AGYW who delayed starting PrEP were 2.89 times more likely to persist (95% confidence interval (CI): 1.52–5.46) and those receiving services via the community-concierge model were 8.7 times (95% CI: 5.44–13.9) more likely to persist (compared to the hybrid model).

**Conclusion:**

Community-based models of PrEP service delivery to AGYW can achieve high PrEP education and HIV testing coverage and moderate PrEP uptake. AGYW-centered approaches to delivering PrEP refills can promote higher persistence.

## Introduction

In sub-Saharan Africa (SSA), adolescent girls and young women (AGYW) are disproportionately impacted by HIV, with 25% of new HIV infections occurring among females aged 15–24 years despite representing just 10% of the population [1]. Daily oral pre-exposure prophylaxis (PrEP) is a biomedical HIV prevention method which is highly protective against HIV infection [2–4]. PrEP is an especially promising prevention strategy for AGYW as it is user-controlled, which may mitigate significant challenges AGYW face in negotiating condom use and utilizing other HIV prevention methods. Beginning with open-label trials and followed by demonstration projects in Kenya and South Africa, PrEP service delivery for AGYW has since expanded to more than 15 countries in SSA, where implementation is largely concentrated in high HIV burden areas [4–7].

Despite this progress, substantial gaps remain in access to PrEP services among AGYW, hindering their uptake of and persistence with PrEP. PrEP service delivery models broadly define the way PrEP services are delivered and to-date, PrEP has largely been delivered via clinic-based models. In these models, PrEP services are often delivered within existing HIV care and treatment clinics where considerable HIV-related stigma can prevent AGYW from seeking and using PrEP [8–10]. Judgmental treatment by healthcare providers (HCP) also acts as a barrier to accessing PrEP; providers sometimes question whether adolescents should be using PrEP or other sexual and reproductive health (SRH) services and/or may refuse to provide such services [11, 12]. Gaps in the quality of healthcare services for adolescents, including insufficient privacy in clinic consultation rooms and waiting areas, long wait times, inconvenient clinic locations, and inaccessible hours (relative to the school day), have also been cited as important barriers for AGYW to accessing and using clinic-based PrEP services [9, 10, 13].

Community-based PrEP service delivery models have been proposed to address these barriers and support AGYW to more readily obtain and use PrEP services [4, 14–17]. Community-based HIV and ART service delivery have demonstrated improved patient outcomes compared to clinic-based delivery models for multiple populations, including adolescents [18–20]. However, evidence from the delivery of PrEP services outside of clinic settings is limited [21]. Understanding the coverage of PrEP services achieved by community-based models (i.e., the proportion of AGYW in high-HIV burden settings using PrEP services such as PrEP education, HIV testing, PrEP initiation, PrEP refills) and measuring factors associated with PrEP uptake and persistence among AGYW accessing services through such models could help to identify successes and gaps in service delivery and inform scale-up in other settings. Given the high rates of early drop-off one-month after PrEP initiation among AGYW reported across PrEP research and programmatic settings, more insight into factors influencing persistence in this early period are particularly needed [15, 22–27].

We evaluated coverage of PrEP services as well as PrEP uptake and early PrEP persistence among AGYW participating in DREAMS (Determined, Resilient, Empowered, AIDS-free, Mentored, and Safe) HIV prevention programming and receiving PrEP through community and hybrid service delivery models in Namibia. We further examined factors associated with PrEP uptake and early persistence to inform future intervention opportunities to promote PrEP use among AGYW.

## Methods

### Program and population

This study draws from PrEP-related service delivery data for AGYW aged 15 to 24 who participated in DREAMS in Namibia’s Khomas and Zambezi regions between December 2018 and September 2019. DREAMS in Namibia is supported by the United States President’s Emergency Plan for AIDS Relief (PEPFAR) with the goal of preventing new HIV infections among high-risk AGYW through community-based provision of a comprehensive package of evidence-based biomedical, behavioral, and structural interventions, including PrEP [6]. DREAMS launched in Namibia in late-2017 and implementation focused on Khomas, Zambezi, and Oshikoto regions due to their higher estimated HIV incidence among AGYW [28]. From 2017 through 2019, DREAMS in Khomas and Zambezi regions was implemented by the International Training and Education Center for Health (I-TECH) in collaboration with the Ministry of Health and Social Services (MoHSS); the Ministry of Education, Arts, and Culture (MoEAC); the Ministry of Gender Equality, Poverty Eradication and Social Welfare; and partner non-governmental organizations Lifeline/Childline of Namibia and Star for Life [29]. In Khomas, DREAMS implementation concentrated within urban and informal settlement areas in and around Windhoek, Namibia’s capital city. In Zambezi, implementation spanned urban and rural areas, including sites along the main transportation corridor in the region.

Upon entry into DREAMS, AGYW were placed into groups based on age (i.e., 9–14, 15–19, and 20–24) and location. Within these groups, AGYW were provided HIV and gender-based violence (GBV) prevention education, sexual and reproductive health (SRH) services (including HIV services), and social services, such as GBV case management. All interventions were delivered in community-based “safe spaces”–judgment-free, safe, and private locations where groups of AGYW attended regular meetings led by a trained peer or near-peer mentor [30]. In the Namibian context, safe spaces included school classrooms; rooms or open spaces within local non-governmental organizations or community center compounds; rooms in technical colleges or universities; adolescent service delivery rooms in health centers; and church meeting rooms. All locations operated as safe spaces only at designated DREAMS meeting or service delivery days and times and were not open on a walk-in basis. Safe spaces were selected through a mapping exercise that involved interviews with AGYW and community members to gather information such as where AGYW would feel most safe to gather (and where not), how far they would be comfortable to travel, and what resources were already available in the location [31]. In December 2018, a DREAMS eligibility assessment tool was introduced at enrollment into DREAMS programming. The tool was a survey consisting of questions about the AGYW’s background (e.g., education, emotional support, financial support), alcohol and substance use, sexual behaviors (e.g., condom use, concurrent partners), and experience(s) of GBV (e.g., physical, emotional, or sexual violence). The tool was self-administered or administered via support from a DREAMS staff member, if requested. Combinations of age and certain responses were used to identify AGYW possibly more vulnerable to HIV infection and/or GBV and, thus, eligible for secondary DREAMS interventions such as economic empowerment programs.

### PrEP service delivery

In Namibia, PrEP for the prevention of HIV was introduced in the 2016 National Antiretroviral (ART) Guidelines [32]. During the study period, PrEP service delivery in DREAMS in Khomas and Zambezi regions was implemented by I-TECH in collaboration with MoHSS, MoEAC, and Star for Life (in Windhoek only). To generate demand for PrEP, flyers with basic information about PrEP (e.g., what is PrEP, who is PrEP for, how do I get PrEP, how do I take PrEP) targeted to young people were designed by the DREAMS program. Flyers were disseminated at DREAMS recruitment events, HIV prevention education sessions, and health services visits. Training on the impact of HIV on AGYW, PrEP, PrEP service delivery, and adolescent-friendly service delivery was conducted with DREAMS and selected MoHSS providers to support the provision of PrEP to AGYW in DREAMS and in MoHSS facilities.

AGYW participating in DREAMS and aged 15–24 received PrEP services via three models–a “community-concierge” model, a “community-fixed” model, and a hybrid “community-clinic” model (Table 1A–1C). Definitions and frameworks for naming and describing PrEP service delivery models vary in practice and in the literature. In this paper, we conceptualize PrEP service delivery models as comprised of four building blocks–when the service is provided, where the service is provided, who is providing the service, and what services are provided [33]. We define these building blocks separately for i) initial PrEP services (education, screening, initiation) and ii) initial follow-up (0–3 months; refills, clinical monitoring, and support). Models were named according to where services were provided.

**Table 1:** a-c. Key components of PrEP Service Delivery Models Implemented within DREAMS Programming in Khomas and Zambezi Regions of Namibia 2018–2019.

a. Community-concierge model														
	INITIAL PrEP SERVICES							INITIAL FOLLOW-UP SERVICES (0–3 MONTHS)						
	Education		Screening			Initiation		Refills			Clinical monitoring		Support	
**WHEN** Service frequency	During prevention education and/or DREAMS health services visit		During any DREAMS health services visit			Same day as screening		1 month after initiation and every 1–3 months thereafter to align with client needs or preferences^8^			1 month following initiation and every 3 months thereafter		Prior to scheduled visit and after missed visit, or at client’s discretion	
**WHERE** Service location	Community-based location, typically a DREAMS safe space^1^		Community-based location, typically a DREAMS safe space^1^			Community-based location, typically a DREAMS safe space^1^		Fixed or mobile location, individually determined for AGYW convenience^9^			Fixed or mobile location, individually determined for AGYW convenience^9^		Phone/SMS	
**WHO** Service provider	DREAMS peer mentor^2^ and health assistant^3^		DREAMS health assistant^3^ and nurse^4^			DREAMS nurse^4^		DREAMS nurse^4^			DREAMS nurse^4^		DREAMS health assistant^3^ and nurse^4^	
**WHAT** Service package	Basic HIV risk reduction, HIV prevention, and PrEP education		Counseling on combination HIV prevention, risk screening, HTS Risk screening by nurse if not completed by health assistant			PrEP offer; use & adherence counseling Blood draw^5^ for CrCl; samples deposited at nearby government health facility and integrated into routine laboratory testing SRH^6^ & social services^7^		Prescription and dispensing of 1–3 months of PrEP medication Screening and mgmt. of side effects; use & adherence counseling; FP method provision and other SRH services as needed			Screening and management of side effects; use & adherence counseling; HTS; repeat CrCl according to guidelines SRH services^6^		Pre-visit reminder and confirmation of convenient visit date, time, and location Reminder call after missed visit Client-led contact with DREAMS nurse for questions or concerns	
**b. Community-fixed model**														
	**INITIAL PrEP SERVICES**						**INITIAL FOLLOW-UP SERVICES (0–3 MONTHS)**							
	Education	Screening		Initiation			Refills		Clinical monitoring			Support		
**WHEN** Service frequency	Initial PrEP service delivery identical across models.						1 month after initiation and every 1–3 months thereafter to align with client needs and preferences^10^		1 month following initiation and every 3 months thereafter			Prior to scheduled visit and after missed visit, or at client’s discretion		
**WHERE** Service location							A set, scheduled rotation of fixed locations (typically DREAMS safe spaces^11^) dependent on provider availability and client preference^12^		A set, scheduled rotation of fixed locations (typically DREAMS safe spaces^11^) dependent on provider availability and client preference^12^			Phone/SMS		
**WHO** Service provider							DREAMS nurse^13^		DREAMS health assistant^14^ and/or DREAMS nurse^13^			DREAMS health assistant^14^ and nurse^13^		
**WHAT** Service package							Provision of 1–3 months of PrEP medication Screening and mgmt. of side effects; use & adherence counseling; FP method provision and other SRH services as needed		Fast-tracked Screening and management of side effects; use & adherence counseling; HTS; repeat CrCl according to guidelines SRH services^15^			Reminder message or call prior to scheduled visit and after missed visit Client-led contact with DREAMS nurse for questions or concerns		
**c. Hybrid community-clinic model**														
	**INITIAL PrEP SERVICES**						**INITIAL FOLLOW-UP SERVICES (0–3 MONTHS)**							
	Education	Screening			Initiation		Refills			Clinical monitoring				Support
**WHEN** Service frequency	Initial PrEP service delivery identical across models.						1 month after initiation and every 3 months thereafter			1 month following initiation and every 3 months thereafter				--
**WHERE** Service location							ART clinic in the MoHSS health facility and ART or clinic pharmacy			ART clinic in the MoHSS health facility				--
**WHO** Service provider							Facility-based ART nurse^17^ and pharmacy assistant/pharmacist			Facility-based health assistant^18^ and/or ART nurse^17^				--
**WHAT** Service package							Prescription and dispensing of PrEP medication Screening and mgmt. of side effects; use & adherence counseling			Screening and management of side effects; use & adherence counseling; HTS; repeat CrCl according to guidelines Provision alongside comprehensive SRH services recommended				--

Abbreviations: CrCl: Creatinine clearance. DREAMS: Determined, Resilient, Empowered, AIDS-free, Mentored, Safe program. FP: family planning. HBsAg: Hepatitis B surface Antigen. HTS: HIV testing services. PrEP: pre-exposure prophylaxis. SMS: short message service. SRH: Sexual and reproductive health.

^1^ Judgment-free, safe, and private locations selected through mapping exercise with AGYW and community members and included school classrooms; rooms or open spaces within local non-governmental organizations or community center compounds; rooms in technical colleges or universities; adolescent service delivery rooms in health centres; and church meeting rooms.

^2^ AGYW peer or near-peer mentor trained to lead regular meetings with groups of AGYW and provide HIV and GBV prevention education.

^3^ Healthcare cadre who provides HIV counseling and testing services in Namibia.

^4^ Adolescent-friendly, primarily female, nurses trained in nurse-initiated management of ART (NIMART) and PrEP.

^5^ Samples stored in a cooler and dropped off at the nearest government health facility at end-of-day for pick-up and processing by the Namibia Institute of Pathology (NIP) laboratory as part of routine laboratory services.

^6^ Sexual and reproductive health (SRH) service package included family planning counseling and method provision, symptomatic screening and treatment for sexually transmitted infections, pregnancy testing (if clinically indicated), and condom provision.

^7^ Social services package was provided by counselors or social workers and included psychosocial support and gender-based violence screening, case management, and referrals. Social services were typically provided on or close to the same day as health services with follow-up services provided as needed.

^8^ Refill frequency determined by client need such as to align with FP method frequency, travel, school holidays, or more convenient for the client for any other reason.

^9^ A community-based location (day and time) individually-tailored through consultation with the client and responding to her real-time needs and preferences including fixed sites (e.g., at a DREAMS safe space, after hours at their secondary school, near their home, at a market, or in a community center), a mobile van temporarily parked outside of one of these fixed community-based sites, or within the client’s home or other preferred location.

^10^ Refill frequency determined by client need such as to align with FP method frequency, travel, school holidays, or more convenient for the client for any other reason.

^11^ Judgment-free, safe, and private locations selected through mapping exercise with AGYW and community members and included school classrooms; rooms or open spaces within local non-governmental organizations or community center compounds; rooms in technical colleges or universities; adolescent service delivery rooms in health centres; and church meeting rooms.

^12^ A small group of DREAMS nurses provided SRH and PrEP services to AGYW at certain DREAMS safe spaces on a set schedule, depending on HCP availability. From this set schedule, AGYW could choose their preferred date/time and location, which may or may not have been where they initiated PrEP.

^13^ Adolescent-friendly, primarily female, nurses trained in nurse-initiated management of ART (NIMART) and PrEP.

^14^ Healthcare cadre who provides HIV counseling and testing services in Namibia.

^15^ Sexual and reproductive health (SRH) service package included family planning counseling and method provision, symptomatic screening and treatment for sexually transmitted infections, pregnancy testing (if clinically indicated), and condom provision.

^16^ Provider may choose to provide refills at different frequencies because of stock availability or concerns about consistent use/adherence.

^17^ NIMART-certified MoHSS nurse providing HIV care and treatment services in the ART clinic within a public health facility who received additional PrEP training.

^18^ Healthcare cadre who provides HIV counseling and testing services in Namibia.

All three models were identical across initial PrEP services–PrEP education, clinical eligibility and HIV risk screening, and PrEP initiation (Table 1A). PrEP education was provided by DREAMS peer mentors during HIV/GBV prevention education sessions and by DREAMS peer mentors, health assistants (a cadre who provides HIV testing and counseling), or nurses during DREAMS health services visits at DREAMS safe spaces. Health assistants or nurses (whichever available) conducted clinical eligibility screening including HIV testing services and HIV risk assessment in the form of client-centered counseling on HIV risk and prevention methods. PrEP was offered in line with MoHSS national guidelines to AGYW aged 15 and older, who tested HIV-negative, who screened at substantial risk for HIV infection or who considered themselves at risk, and did not have clinical contraindications [32, 34]. PrEP initiation occurred the same-day as HTS and risk screening. Initial PrEP services were provided as part of a package of SRH services which included HIV testing services (HTS), syndromic screening for sexually transmitted infections (STIs), and contraceptive counseling and service provision. For those who were clinically eligible and accepted PrEP, nurses prescribed PrEP and provided additional counseling on use, adherence, and follow-up and confirmed with AGYW their next visit date. PrEP was dispensed and venous blood for required creatinine clearance (CrCl) was drawn by the prescribing nurse. Samples were dropped off at a public health facility at the end of the day for processing.

The three models, however, differed in the delivery of initial follow-up services–refills, clinical monitoring, and support. In the “community-concierge” model (Table 1A), DREAMS nurses delivered PrEP refills and clinical monitoring at community-based locations which were individually-tailored to the AGYW’s real-time needs and preferences. Locations included fixed sites (e.g., at a DREAMS safe space, after hours at their secondary school, near their home, at a market, or in a community center), a mobile van temporarily parked outside of one of these fixed community-based sites, or within the AGYW’s home or other preferred location. DREAMS providers called AGYW prior to their anticipated refill date to coordinate a convenient visit date, time, and location. Refills occurred one month after initiation and every one to three months thereafter depending on the AGYW’s needs or preferences (e.g., to align with contraceptive method provision or accommodate travel during school holidays). Refills were delivered alongside screening and management of side effects, adherence counseling, and SRH services (e.g., contraception method provision, STI screening). One month after initiation and every three months thereafter, DREAMS nurses also conducted clinical monitoring for PrEP (HIV test and repeat laboratory tests according to guidelines). DREAMS HCPs conducted SMS or phone call reminders prior to scheduled refill and follow-up visits and after missed visits. AGYW could call or message a DREAMS nurse-managed phone with questions or concerns.

In the “community-fixed” model (Table 1B), DREAMS nurses delivered PrEP refills and clinical follow-up services to AGYW via scheduled visits at fixed community-based locations (e.g., DREAMS safe spaces). AGYW could select their preferred visit location, date, and time from a set, scheduled rotation. At each fixed site, HCP were only available during scheduled day and time (e.g., sites were not ‘drop-in’). All other aspects of service delivery for refills, clinical monitoring, and support in the community-fixed model mirrored the community-concierge model.

Lastly, in the hybrid “community-clinic” model (Table 1C), AGYW were referred to their preferred public health facility for PrEP refills and clinical monitoring which were provided by MoHSS clinic-based HCPs. NIMART (nurse-initiated management of antiretroviral therapy) nurses, typically located in the ART clinic, prescribed refills one month after initiation and every three months thereafter. Refill visits were delivered alongside screening and management of side effects and adherence counseling. PrEP was dispensed at the ART pharmacy or clinic pharmacy where no separate ART pharmacy existed. Nurses also conducted clinical monitoring for PrEP every three months. AGYW receiving services through the hybrid community-clinic model did not routinely receive reminder calls or messages, however they could call or message a DREAMS nurse-managed phone with questions or concerns.

Model choice was geographically and temporally limited as implementation was phased over the study period. However, AGYW could choose their preferred PrEP service delivery model from all currently implemented models at their PrEP initiation.

### Data collection

For this study, we used two routine programmatic data sources–electronic DREAMS participant records and paper-based MoHSS PrEP client records of AGYW aged 15–24 –in which individual-level data were documented as part of routine DREAMS program implementation and PrEP service delivery. We abstracted data on socio-demographic information (age, location, relationship type), structural and behavioral HIV risk factors at DREAMS entry (dropped out of school before grade 12, food insecurity, ever-experienced GBV, current pregnancy, alcohol and/or drug use, number of sexual partners in past 12 months, age-disparate partner(s), concurrent partners, condom use, transactional sex) and details of health service uptake and delivery (PrEP education, HTS, and PrEP initiation) from DREAMS participant records. From PrEP client records, we abstracted routinely collected information on client characteristics at PrEP initiation (previous PrEP use, any documented HIV risk factors) and service provision data from follow-up PrEP visits (scheduled and actual visit date, PrEP prescription). Individual-level data were linked across sources using the unique DREAMS participant ID.

### Statistical analysis

We evaluated coverage of PrEP services using a cascade reflective of five steps of PrEP delivery in this setting [22]. Specifically, we described proportions of AGYW 15–24 at each step in the cascade with the denominator starting as the number of AGYW participating in DREAMS and each next step using the numerator of the previous step as its denominator. We excluded AGYW already on PrEP at entry into DREAMS (n = 32). Cascade steps included: 1) received PrEP education; 2) tested for HIV; 3) tested HIV-negative; 4) initiated PrEP; and 5) persisted with PrEP at month one, defined as receiving PrEP refill prescription 15–44 days after PrEP initiation (with the denominator restricted to those who initiated PrEP 45 days or more before the end of the study period). Cascade step five was further stratified by delivery model with differences between models tested using a chi-squared test.

We separately explored factors associated with PrEP uptake and early PrEP persistence among AGYW aged 15–24 as our primary outcomes of interest. ‘PrEP uptake’ was defined as the proportion of AGYW who initiated PrEP among those who received PrEP education and tested HIV-negative. ‘Early PrEP persistence’ was defined as the proportion of AGYW who persisted with PrEP at month one among those expected for a month one visit during the study period. In univariable models, we explored each potential correlate of PrEP uptake and early persistence. We then used backwards elimination to select factors for inclusion in each multivariable model by eliminating variables by highest p-value until a threshold of p < 0.2 was met. We used generalized estimating equations with log links and Poisson distributions to estimate prevalence ratios (PR) while accounting for clustering by DREAMS entry group. Analyses were conducted using RStudio (version 4.0.5) and the *geepack* package [35–37].

### Ethical review

This project was reviewed and approved by ethics committees at the U.S. Centers for Disease Control and Prevention, the Namibian Ministry of Health and Social Services, and the University of Washington. Prior to participation in DREAMS, AGYW provided written informed consent and written parental consent was obtained from parents of AGYW aged 9–19 years. A waiver of consent was obtained for the review of DREAMS participant records and PrEP client records, which were fully anonymized before being accessed by the study team.

## Results

### Service coverage along a PrEP cascade

**Fig 1:**
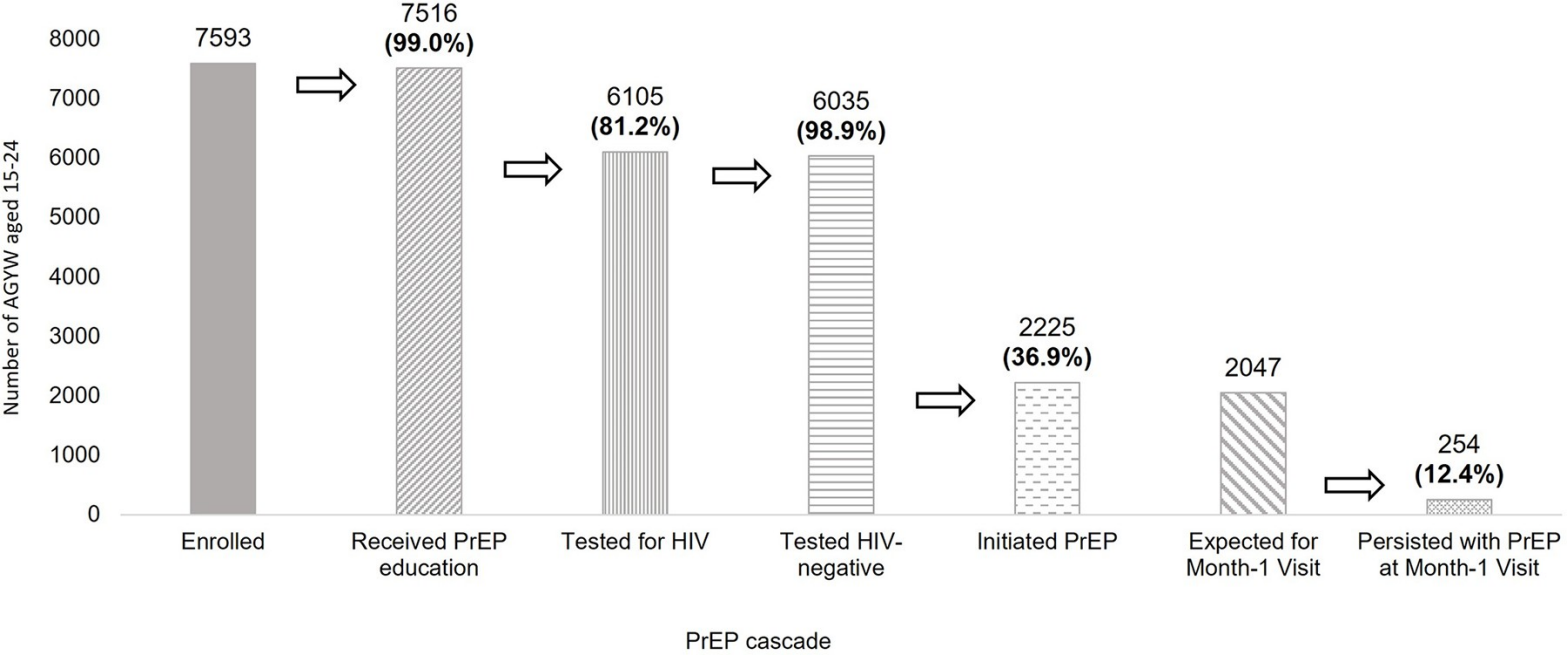
PrEP cascade, overall.

**Fig 2:**
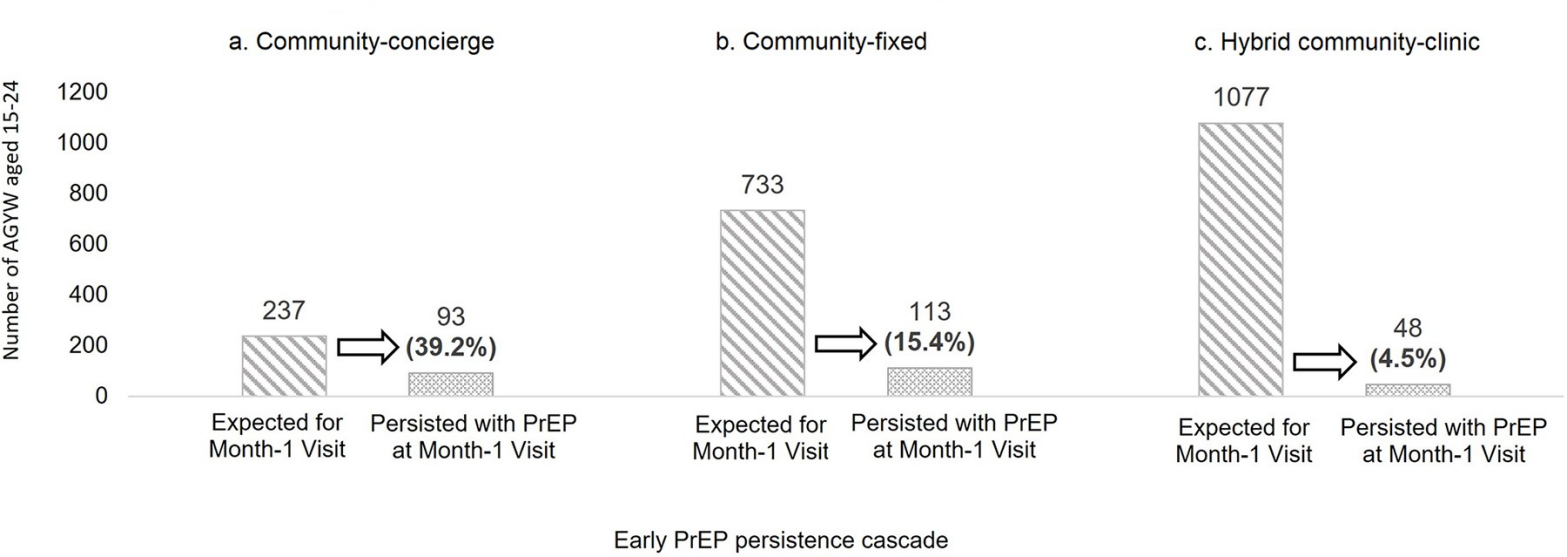
**a-c.** Early PrEP persistence cascade, by service delivery model.

Between December 1, 2018 and September 30, 2019, 7593 AGYW aged 15–24 enrolled in DREAMS HIV prevention programming and were not already using PrEP at enrollment. The median follow-up time for participants was 142 days (min = 0, max = 301). Of enrolled AGYW, 7516 (99.0%) received PrEP education and 6105 (81.2%) received HIV testing services with 6035 (98.9%) testing HIV-negative (Fig 1). Among AGYW who tested HIV-negative, 2225 (36.9%) initiated PrEP. Of the 2047 AGYW expected for a month one visit within the study period, 254 (12.4%) persisted with PrEP. Of the 328 AGYW who did not persist with PrEP one month after initiation and had at least six months post-initiation time within the study period, 24 (7.3%) restarted PrEP. Three (0.1%) AGYW who initiated PrEP during the study period later tested positive for HIV infection; two clients received an HIV-positive test result at their month-one follow-up visit and one client received an HIV-positive test result at a follow-up visit four months post-initiation after having received 90-days PrEP prescription at their month-one visit. We found significant differences in early PrEP persistence by service delivery model, with 39.2% of AGYW persisting on PrEP at one-month post-initiation in the community-concierge, 15.4% in the community-fixed, and 4.5% in the hybrid community-clinic model (Fig 2A–2C, p<0.001).

### Factors associated with PrEP uptake

Overall, 5742 AGYW were eligible for PrEP, defined as those who received PrEP education and tested HIV-negative, and were included in our analysis of factors associated with PrEP uptake. We excluded AGYW with missing data for all structural, biological, and behavioral HIV risk factors assessed at DREAMS entry (n = 293); missing all data were not associated with PrEP uptake (p = 0.81). As shown in Table 2, most AGYW were aged 15–19 years (n = 3374; 58.8%), located in Khomas Region (n = 4305; 75.0%), and classified at higher risk for HIV infection and eligible for secondary DREAMS interventions (n = 4653; 81.0%). Among AGYW, 24.7% (n = 1396) reported having had dropped out of school before grade 12, 32.6% (n = 1850) reported food insecurity, and 26.2% (n = 1499) reported ever experiencing any type of GBV. Intermittent or no condom use was the most commonly reported behavioral risk factor (n = 2316; 58.9%). PrEP uptake among AGYW who were not missing all data on structural, biological, and behavioral HIV risk factors assessed at DREAMS entry and who tested HIV-negative (n = 5742) was 36.8% (n = 2115).

**Table 2:** Socio-demographic characteristics and HIV risk factors among AGYW eligible for PrEP^1^ and associations with PrEP uptake.

Characteristic		Proportion of AGYW among all eligible for PrEP^1^ (N = 5742)	Proportion of initiators within the risk category	Prevalence ratio (95% CI)	p-value	Adjusted prevalence ratio (95% CI)	p-value
Eligible for secondary DREAMS interventions^2^		4653 (81.0%)					
Initiated PrEP^3^		2115 (36.8%)					
*Socio-demographic characteristics*							
Age Group							
	15–19	3374 (58.8%)	1013/3374 (30.0%)	1	--	1	--
	20–24	2368 (41.2%)	1103/2369 (46.6%)	1.56 (1.37–1.77)	**<0.001**	1.08 (1.00–1.16)	0.051
Geographic location							
	Khomas Region	4304 (75.0%)	1410/4305 (32.8%)	1	--	1	--
	Zambezi Region	1438 (25.0%)	706/1438 (49.1%)	1.95 (1.55–2.45)	**<0.001**	1.31 (1.10–1.56)	**0.003**
Relationship type							
	A main partner^4^	3398/5640 (60.2%)	1625/3398 (47.8%)	1	--	1	--
	No partner	2092/5640 (37.1%)	398/2092 (19.0%)	0.43 (0.37–0.50)	**<0.001**	0.81 (0.71–0.91)	**<0.001**
	Only casual partner(s)	150/5640 (2.7%)	56/150 (37.3%)	0.87 (0.73–1.03)	0.109	0.95 (0.78–1.15)	0.608
*Structural HIV risk factors*							
Dropped out of school^5^							
	No	4259/5655 (75.3%)	1286/4295 (29.9%)	1	--	1	--
	Yes	1396/5655 (24.7%)	785/1396 (56.2%)	1.73 (1.52–1.96)	**<0.001**	1.15 (1.04–1.28)	**0.006**
Food insecure^6^							
	No	3830/5680 (67.4%)	1198/3830 (31.3%)	1	--	1	--
	Yes	1850/5680 (32.6%)	893/1850 (48.3%)	1.36 (1.24–1.49)	**<0.001**	1.15 (1.06–1.24)	**<0.001**
Ever experienced GBV^7^							
	No	4227/5726 (73.8%)	1509/4227 (35.7%)	1	--		
	Yes	1499/5726 (26.2%)	603/1499 (40.2%)	1.13 (1.05–1.21)	**0.001**		
*Clinical and behavioral HIV risk factors*							
Currently pregnant							
	No	5229/5384 (97.1%)	1947/5229 (37.2%)	1	--		
	Yes	155/5384 (2.9%)	84/155 (54.2%)	1.33 (1.12–1.58)	**0.001**		
Alcohol and/or drug use^8^							
	No	4914/5333 (92.1%)	1805/4915 (36.7%)	1	--	1	
	Yes	419/5333 (7.9%)	169/419 (40.2%)	1.19 (1.05–1.36)	**0.008**	1.09 (.096–1.23)	0.181
Two or more partners past 12 months							
	No	4728/5383 (87.8%)	1682/4728 (35.6%)	1	--	1	--
	Yes	655/5383 (12.2%)	337/655 (51.5%)	1.50 (1.37–1.65)	**<0.001**	1.14 (1.04–1.24)	**0.003**
Last partner more than 5 years older							
	No	4503/5334 (84.4%)	1556/4503 (34.6%)	1	--		
	Yes	831/5334 (15.6%)	447/831 (53.8%)	1.44 (1.28–1.62)	**<0.001**		
Ever had concurrent partners							
	No	3626/3950 (91.8%)	1710/3626 (47.2%)	1	--	1	--
	Yes	324/3950 (8.2%)	181/324 (55.9%)	1.21 (1.11–1.32)	**<0.001**	1.13 (1.02–1.24)	**0.017**
Intermittent or no condom use							
	No	1618/3934 (41.4%)	646/1618 (39.9%)	1	--	1	--
	Yes	2316/3934 (58.9%)	1247/2316 (53.8%)	1.28 (1.17–1.40)	**<0.001**	1.22 (1.13–1.33)	**<0.001**
Ever had transactional sex^9^							
	No	5189/5443 (95.3%)	1904/5189 (36.7%)	1	--	1	--
	Yes	254/5443 (4.7%)	170/254 (66.9%)	1.69 (1.48–1.93)	**<0.001**	1.22 (1.07–1.39)	**0.002**

Data are presented as n (%) or, when data were missing for some AGYW, n/N (%).

Statistical significance is evaluated at p values of ≤ 0.05 and statistically significant values are in bold.

^1^ Analysis restricted to all AGYW who were eligible for PrEP (received PrEP education and tested HIV negative as a proxy).

^2^ Classification at higher risk for HIV infection and eligible for secondary DREAMS interventions (e.g., economic strengthening) based on a composite score from select, self-reported structural, clinical, and behavioral HIV risk factors.

^3^ Initiated PrEP during a DREAMS health visit.

^4^ Has a main partner with or without additional casual partners.

^5^ Dropped out of school before grade 12.

^6^ No food in house due to lack of funds one or more times in the past month.

^7^ Has ever experienced emotional, physical, or sexual gender-based violence.

^8^ Alcohol or drug use that interferes with studies, work, hobbies, or puts one in dangerous situations.

^9^ Has ever exchanged sex or been in a relationship for goods, housing, or other benefits.

Multivariable analysis revealed several socio-demographic, structural, and behavioral risk factors associated with PrEP uptake (Table 2). PrEP uptake was more likely among AGYW in Zambezi (aPR: 1.31; 95% CI: 1.10–1.56) as well as among those who reported dropping out of school before grade 12 (aPR: 1.15; 95% CI: 1.04–1.28), food insecurity (aPR: 1.15; 95% CI: 1.06–1.24), intermittent or no condom use (aPR: 1.22; 95% CI: 1.13–1.33), having two or more partners in the past 12 months (aPR: 1.14; 95% CI: 1.04–1.24), ever having concurrent partners (aPR: 1.13; 95% CI: 1.02–1.24), or ever having transactional sex (aPR: 1.22; 95% CI: 1.07–1.39). PrEP uptake was less likely among AGYW who reported no current partner (aPR: 0.81; 95% CI: 0.71–0.91).

### Factors associated with early PrEP persistence

Overall, 1953 AGYW were expected for their month one visit by the end of the study period and included in our analysis of factors associated with early PrEP persistence. We excluded AGYW who were missing data for all structural, biological, and behavioral HIV risk factors assessed at DREAMS entry (n = 94); missing all data were not associated with early PrEP persistence (p = 0.45). As shown in Table 3, 47% of AGYW (n = 917) were aged 15–19 years. Very few AGYW (n = 53; 2.7%) delayed starting PrEP, defined as starting PrEP at the second or later health service visit. Among AGYW, 53.7% (n = 1049) received PrEP services via the hybrid community-clinic model, 34.3% (n = 669) via the community-fixed model, and 12.0% (n = 235) via the community-concierge model. At PrEP initiation, 4.4% of AGYW (n = 86) reported having an HIV-positive partner, whereas 44.2% (n = 864) reported their partner’s HIV status as unknown. Overall, 33.2% of AGYW (n = 648) considered themselves at risk, but did not disclose any specific risk factors during risk screening. Early PrEP persistence among AGYW who were not missing all data on structural, biological, and behavioral HIV risk factors assessed at DREAMS entry and who were expected for their month one visit (n = 1953) was 12.3% (n = 240).

**Table 3:** Socio-demographic characteristics, HIV risk factors, PrEP service uptake and delivery characteristics among AGYW initiated on PrEP^1^ and Associations with PrEP early persistence.

Characteristic		Proportion of AGYW among all PrEP initiators^1^ (N = 1953)	Proportion of persistors within the risk category	Prevalence ratio (95% CI)	p-value	Adjusted prevalence ratio (95% CI)	p-value
Eligible for secondary DREAMS interventions^2^		1773 (90.8%)					
Persisted with PrEP^3^		240 (12.3%)					
*Socio-demographic characteristics*							
Age Group							
	15–19	917 (47.0%)	116/917 (12.6%)	1	--	1	--
	20–24	1036 (53.0%)	124/1036 (12.0%)	0.97 (0.74–1.27)	0.809	1.28 (1.02–1.62)	**0.036**
Geographic location							
	Khomas Region	1314 (67.3%)	144/1314 (11.0%)	1	--		
	Zambezi Region	639 (32.7%)	96/639 (15.0%)	1.45 (0.93–2.25)	0.103		
Relationship type							
	A main partner^4^	1512/1919 (78.8%)	183/1512 (12.1%)	1	--	1	--
	No partner	352/1919 (18.3%)	43/352 (12.2%)	(0.74–1.44)	0.861	0.92 (0.67–1.27)	0.621
	Only casual partner(s)	55/1919 (2.9%)	10/55 (18.2%)	1.58 (1.01–2.48)	**0.047**	1.59 (1.07–2.38)	**0.023**
*Structural HIV risk factors*							
Dropped out of school^5^							
	No	1179/1914 (61.6%)	157/1179 (13.3%)	1	--		
	Yes	735/1914 (38.4%)	80/735 (10.9%)	0.73 (0.51–1.04)	0.084		
Food insecure^6^							
	No	1109/1929 (57.5%)	134/1109 (12.1%)	1	--		
	Yes	820/1929 (42.5%)	105/820 (12.8%)	1.02 (0.78–1.33)	0.893		
Ever experienced GBV^7^							
	No	1399/1950 (71.7%)	163/1399 (11.7%)	1	--		
	Yes	551/1950 (28.3%)	76/551 (13.8%)	1.19 (0.87–1.63)	0.280		
*Clinical and behavioral HIV risk factors*							
Currently pregnant							
	No	1795/1877 (95.6%)	221/1795 (12.3%)	1	--		
	Yes	82/1877 (4.4%)	9/82 (11.0%)	0.88 (0.44–1.74)	0.709		
Alcohol and/or drug use^8^							
	No	1670/1821 (91.7%)	214/1670 (12.8%)	1	--	1	
	Yes	151/1821 (8.3%)	11/151 (7.3%)	0.62 (0.39–1.00)	**0.049**	0.61 (0.35–1.05)	0.072
Two or more partners in past 12 months							
	No	1555/1866 (83.3%)	194/1555 (12.5%)	1	--		
	Yes	311/1866 (16.7%)	37/311 (11.9%)	0.96 (0.64–1.46)	0.862		
Last partner more than 5 years older							
	No	1438/1856 (77.5%)	178/1438 (12.4%)	1	--		
	Yes	418/1856 (22.5%)	45/418 (10.8%)	0.99 (0.73–1.34)	0.945		
Ever had concurrent partners							
	No	1588/1755 (90.5%)	200/1588 (12.6%)	1	--		
	Yes	167/1755 (9.5%)	19 /167 (11.4%)	0.92 (0.61–1.38)	0.690		
Intermittent or no condom use							
	No	592/1756 (33.7%)	84/592 (14.2%)	1	--		
	Yes	1164/1756 (66.3%)	138/1164 (11.9%)	0.82 (0.61–1.09)	0.171		
Ever had transactional sex^9^							
	No	1756/1913 (91.8%)	222/1756 (12.6%)	1	--		
	Yes	157/1913 (8.2%)	15/157 (9.6%)	0.75 (0.52–1.10)	0.145		
*HIV risk factors documented at PrEP initiation*							
Partner(s) living with HIV							
	No	1867 (95.6%)	224/1867 (12.0%)	1	--		
	Yes	86 (4.4%)	16/86 (18.6%)	1.42 (0.89–2.28)	0.145		
Partner(s) HIV status unknown							
	No	1089 (55.8%)	141/1089 (12.9%)	1	--	1	
	Yes	864 (44.2%)	99/864 (11.5%)	0.85 (0.66–1.08)	0.180	0.72 (0.54–0.96)	**0.025**
Considers self at risk, only^10^							
	No	1304 (66.8%)	166/1304 (12.7%)	1	--	1	
	Yes	649 (33.2%)	74/649 (11.4%)	0.96 (0.68–1.35)	0.806	0.77 (0.54–1.09)	0.134
*PrEP uptake and service delivery characteristics*							
Delayed starting PrEP^11^							
	No	1900 (97.3%)	221/1900 (11.6%)	1	--	1	--
	Yes	53 (2.7%)	19/53 (35.8%)	2.85 (1.29–6.29)	**0.010**	2.89 (1.52–5.46)	**0.001**
Service delivery model							
	Hybrid community-clinic	1049 (53.7%)	47/1049 (4.5%)	1	--	1	--
	Community-concierge	235 (12.0%)	92/235 (39.1%)	8.61 (5.45–13.6)	**<0.001**	8.70 (5.44–13.9)	**<0.001**
	Community-fixed	669 (34.3%)	101/669 (15.1%)	3.32 (1.99–5.56)	**<0.001**	2.97 (1.80–4.90)	**<0.001**

Data are presented as n (%), or, when data were missing for some AGYW, n/N (%).

Statistical significance is evaluated at p values of ≤ 0.05 and statistically significant values are in bold.

^1^ Analysis restricted to AGYW who initiated PrEP and were expected for their month one visit during the study period.

^2^ Classification at higher risk for HIV infection and eligible for secondary DREAMS interventions (e.g., economic strengthening) based on a composite score from select, self-reported structural, clinical, and behavioral HIV risk factors.

^3^ Received a PrEP refill 15–45 days after initiation.

^4^ Has a main partner with or without additional casual partners.

^5^ Dropped out of school before grade 12.

^6^ No food in house due to lack of funds one or more times in the past month.

^7^ Has ever experienced emotional, physical, or sexual gender-based violence.

^8^ Alcohol or drug use that interferes with studies, work, hobbies, or puts one in dangerous situations.

^9^ Has ever exchanged sex or been in a relationship for goods, housing, or other benefits.

^10^ Only HIV risk factor documented on the PrEP client record is ‘Considers self at risk’.

^11^ Started PrEP at the second or later health services visit.

Multivariable analysis found early PrEP persistence to be 8.7 times more likely among AGYW receiving services in the community-concierge model (aPR: 8.70; 95% CI: 5.44–13.9) and 2.97 times more likely in the community-fixed model (aPR: 2.97; 95% CI: 1.80–4.90) compared to the hybrid community-clinic model (Table 3). In addition, AGYW who delayed PrEP start were 2.89 times more likely to persist one-month after initiation (aPR: 2.89; 95% CI: 1.52–5.46) than those who started on their first health services visit. Early persistence was also more likely among AGYW aged 20–24 or who reported only having casual partners (compared to a main partner). Having a partner with unknown HIV status was the only factor negatively associated with early persistence.

## Discussion

We examined PrEP-related service coverage and evaluated PrEP uptake and persistence among AGYW participating in DREAMS and receiving PrEP via three different service delivery models in two regions in Namibia. PrEP services delivered within DREAMS community-based HIV prevention programming achieved moderate PrEP uptake. PrEP persistence one month after initiation was low overall, however differences by service delivery model were significant, with rates of early persistence substantially and significantly higher among AGYW receiving services in the community-concierge model and the community-fixed model compared with the hybrid community-clinic model.

In all three models, initial PrEP services (e.g., education, screening, initiation) were delivered identically, integrated within community-based HIV prevention programming for AGYW. Our results suggest this approach may support high coverage of initial PrEP services and elicit higher rates of PrEP uptake among AGYW than have been observed through other delivery approaches in SSA. In Kenya, PrEP uptake was only 4% among AGYW receiving services in family planning clinics and 21.7% among women and girls aged 15 and older receiving services in maternal and child health clinics [25, 38]. In the SEARCH trial, 19% of AGYW who tested HIV-negative and were assessed to be at elevated risk of HIV initiated PrEP at community-based health fairs and facilities in rural communities in Kenya and Uganda [39]. Factors that may have contributed to the higher uptake observed in our study include the targeting of AGYW at higher risk for HIV for participation in DREAMS, the DREAMS peer/near-peer mentoring and ‘safe space’ approach where AGYW can build supportive social networks, the use of adolescent-friendly service approaches and service providers, and the concurrent participation in HIV/GBV prevention education, which may build awareness and knowledge of one’s HIV risk and prevention options [40].

The early drop-off rates in our study are similar to those found elsewhere in SSA. Several studies have shown low rates of early PrEP persistence (31–38%) among AGYW receiving services through clinic, mobile clinic, and community-based models in Kenya and South Africa [22, 25–27]. In Namibia, the nascency of the PrEP program and its data systems may have contributed to the low persistence seen across all three models. No clinical trials, open label studies, demonstration projects, or awareness or demand creation campaigns were conducted prior to or concurrent with this study in Namibia. Thus, PrEP awareness was very low among AGYW as well as within their communities, contributing to social and internalized stigma surrounding PrEP use that may have negatively impacted persistence [41]. Data from the United States has shown annual increases in the proportions of clients refilling PrEP prescriptions from PrEP introduction in 2012 through 2017, suggesting a link between greater widespread community awareness and acceptance of PrEP and increases in persistence over time which may be generalizable to other contexts and populations [42, 43]. In addition, the lack of a data system that would enable linkage of client PrEP records across service delivery locations meant that AGYW who returned for PrEP refill to any public or community-based clinic outside of this study would not have been captured.

More than 80% of AGYW in the study were classified as higher risk for HIV and eligible for secondary DREAMS interventions, yet only 37% initiated PrEP. After controlling for behavioral risk factors, AGYW who had dropped out of school before grade 12, were food insecure, or living in a region with high HIV prevalence and incidence among girls and women (Zambezi) were more likely to initiate PrEP, which suggests AGYW may perceive their HIV risk or make PrEP use decisions based not only on their own and their partners’ sexual behaviors but also based on their individual socio-economic circumstances and their awareness of local area-level prevalence or incidence [28, 44, 45]. The inclusion of information about structural HIV risk and local HIV prevalence or incidence in PrEP decision support tools may promote PrEP uptake by helping AGYW recognize broader factors that contribute to their HIV risk as well as supporting more informed prevention choices [46, 47].

Research has shown that in the first months after initiation AGYW may be in a period of adjustment, considering if PrEP is really right for them [10]. Examining PrEP persistence in this early period and in a real-world PrEP delivery context is particularly important to gain an understanding of who is truly interested in and able to continue PrEP after a period of initial experimentation. In our study, we found early PrEP persistence was highly correlated with service delivery model with AGYW receiving services through the community-concierge model 8.70 times more likely to persist and the community-fixed model 2.97 times more likely to persist compared to the hybrid community-clinic model. This finding indicates that PrEP service delivery responsive to AGYWs’ real-time needs and constraints can support higher rates of persistence. Both models featured provider continuity, where the same DREAMS adolescent-friendly providers delivered all PrEP services. Such continuity, particularly where trusting relationships are formed with adolescent-friendly providers, may facilitate PrEP persistence among AGYW [10, 48, 49]. Both models also featured flexibility in service location, where an individual initiates PrEP in one location and receives refills in a different location. This flexibility in service delivery, when client-led and client-centered, supports higher rates of persistence [49, 50]. Where differences in service location are provider- or model-led, as to some extent in the community-fixed model and completely in the hybrid community-clinic model, it may introduce barriers to persistence, as was also experienced by AGYW receiving PrEP services through a mobile clinic in South Africa [48]. Interventions such as tailored counseling or peer navigation may be needed to support AGYW as they transition between community- and clinic-based PrEP service locations over time, especially given their high mobility and in order to bring PrEP for AGYW to scale [10, 40, 48, 51].

Early PrEP persistence was also more likely among AGYW who delayed starting PrEP until their second or later health services visit. Despite being a user-controlled method, qualitative research has highlighted that AGYW may delay starting PrEP in order to discuss and obtain support for PrEP use from their peers, parents or partners and that positive disclosure experiences and outcomes can be leveraged to support continued PrEP use whereas negative experiences and outcomes can lead to PrEP discontinuation [10, 52, 53]. Integration of disclosure support within PrEP counseling for those who would like to discuss their PrEP use or intended use with a peer, family member, caregiver, or partner could help AGYW obtain the emotional and financial support needed to persist with PrEP [52, 54, 55].

The strength of our study includes the use of routine programmatic data to evaluate PrEP-related service coverage, measure key PrEP outcomes, and identify factors associated with these outcomes among AGYW receiving services through community and hybrid community-clinic delivery models in real-world programmatic settings. In addition, this study is one of few to examine associations between structural HIV risk and service delivery related factors and PrEP outcomes [21]. Our study, however, has some limitations. Broadly, programmatic data are often underused for research due to concerns of incompleteness and inaccuracy, though we applied several strategies to improve PrEP data quality during program implementation [56]. Model selection was likely biased by geographic location, age, and school status as the service delivery models available differed by region and delivery preferences among AGYW varied by age and school status. However, as a real-world implementation project these results may simulate how these PrEP delivery options might be offered and selected in resource-limited settings. Regarding early persistence, we relied on PrEP prescription data, as PrEP dispensing data were not available, and assumed that all AGYW prescribed PrEP were dispensed PrEP in-room or picked up their refill from the pharmacy. Our ability to make comparisons and conclusions regarding the performance of service delivery models across studies is limited given variability in the reporting of PrEP cascade components, lack of model specificity, differences in service model maturity, and inconsistent definitions and measures of uptake and persistence across studies [7, 42, 57]. Structural and behavioral risk factors included in this study were self-reported at the time of DREAMS entry, thus, underreporting may have occurred if AGYW were reluctant to share this sensitive and private information or factors may have been misclassified if they changed over time.

## Conclusion

PrEP is an important HIV prevention strategy for AGYW and there is an urgent need to identify and scale more person-centred and responsive PrEP service delivery models and strategies that will meet the needs and preferences of AGYW more effectively and comprehensively. Clinic-based delivery models, however, often present barriers for AGYW in accessing and using PrEP. Our study identifies several opportunities to promote PrEP uptake and persistence through community-based and hybrid PrEP service delivery models. First, programs could consider integrating PrEP services within community-based HIV prevention programming for AGYW to increase their awareness of and access to PrEP services. Second, given the mobility and unpredictability AGYW face in their daily lives, programs could consider community-based service delivery models that employ individualized approaches to the delivery of PrEP refills and follow-up services responsive to client’s real-time needs and constraints as was done in the community-concierge model here. Third, inclusion of information on structural HIV risk as well as area-level HIV prevalence or incidence, in addition to behavioral HIV risk, in PrEP decision support tools could be used to generate a broader understanding of clients’ vulnerability to HIV infection and promote uptake of PrEP or other prevention options among AGYW. Lastly, recognizing that models combining community-based, clinic-based, and other innovative delivery modalities (e.g., pharmacy, telehealth) may be needed to provide PrEP services at scale, programs could consider strategies such as tailored counseling or navigation support to prepare and support clients to receive services across different providers and service locations promoting PrEP persistence as users access PrEP via different service delivery modalities over time.

## Supporting information

S1 Dataset Minimal data set. (XLSX)
